# Comparison of Novel Volumetric Microperimetry Metrics in Intermediate Age-Related Macular Degeneration: PINNACLE Study Report 3

**DOI:** 10.1167/tvst.12.8.21

**Published:** 2023-08-25

**Authors:** Philipp Anders, Ghislaine L. Traber, Maximilian Pfau, Sophie Riedl, Ahmed M. Hagag, Hanna Camenzind, Julia Mai, Rebecca Kaye, Hrvoje Bogunović, Lars G. Fritsche, Daniel Rueckert, Ursula Schmidt-Erfurth, Sobha Sivaprasad, Andrew J. Lotery, Hendrik P. N. Scholl

**Affiliations:** 1 Institute of Molecular and Clinical Ophthalmology Basel, Basel, Switzerland; 2 Department of Ophthalmology, University of Basel, Basel, Switzerland; 3 Ophthalmology Unit, Centro Hospitalar e Universitário de Coimbra (CHUC), Coimbra, Portugal; 4 AIBILI, Association for Innovation and Biomedical Research on Light and Image, Coimbra, Portugal; 5 Laboratory for Ophthalmic Image Analysis, Department of Ophthalmology and Optometry, Medical University of Vienna, Vienna, Austria; 6 Moorfields Eye Hospital NHS Foundation Trust, London, UK; 7 Institute of Ophthalmology, University College London, London, UK; 8 Boehringer Ingelheim Limited, Bracknell, UK; 9 Faculty of Medicine, University of Southampton, Southampton, UK; 10 Center for Statistical Genetics, Department of Biostatistics, University of Michigan School of Public Health, Ann Arbor, MI, USA; 11 Imperial College London, London, UK; 12 Klinikum rechts der Isar, TU Munich, Munich, Germany

**Keywords:** age-related macular degeneration (AMD), intermediate AMD, microperimetry, hill-of-vison (HOV), volumetric assessment

## Abstract

**Purpose:**

To investigate and compare novel volumetric microperimetry (MP)–derived metrics in intermediate age-related macular degeneration (iAMD), as current MP metrics show high variability and low sensitivity.

**Methods:**

This is a cross-sectional analysis of microperimetry baseline data from the multicenter, prospective PINNACLE study (ClinicalTrials.gov NCT04269304). The Visual Field Modeling and Analysis (VFMA) software and an open-source implementation (OSI) were applied to calculate MP-derived hill-of-vison (HOV) surface plots and the total volume (VTOT) beneath the plots. Bland–Altman plots were used for methodologic comparison, and the association of retinal sensitivity metrics with explanatory variables was tested with mixed-effects models.

**Results:**

In total, 247 eyes of 189 participants (75 ± 7.3 years) were included in the analysis. The VTOT output of VFMA and OSI exhibited a significant difference (*P* < 0.0001). VFMA yielded slightly higher coefficients of determination than OSI and mean sensitivity (MS) in univariable and multivariable modeling, for example, in association with low-luminance visual acuity (LLVA) (marginal *R*^2^/conditional *R*^2^: VFMA 0.171/0.771, OSI 0.162/0.765, MS 0.133/0.755). In the multivariable analysis, LLVA was the only demonstrable predictor of VFMA VTOT (*t*-value, *P*-value: −7.5, <0.001) and MS (−6.5, <0.001).

**Conclusions:**

The HOV-derived metric of VTOT exhibits favorable characteristics compared to MS in evaluating retinal sensitivity. The output of VFMA and OSI is not exactly interchangeable in this cross-sectional analysis. Longitudinal analysis is necessary to assess their performance in ability-to-detect change.

**Translational Relevance:**

This study explores new volumetric MP endpoints for future application in therapeutic trials in iAMD and reports specific characteristics of the available HOV software applications.

## Introduction

Age-related macular degeneration (AMD) is the most common cause of blindness in advanced age, with an estimated prevalence of nearly 300 million by 2040.^1^ AMD often comes with a high burden for affected patients, because it deranges the central retina, which is responsible for detailed, high-resolution vision. Compared to persons with normal aging changes, patients with intermediate AMD (iAMD) have an increased risk of progression to late AMD.^2^ To evaluate and predict the trajectory from iAMD to late AMD stages, adequate endpoints are a prerequisite. To this end, elaborate analyses of disease stages and progression based on novel optical coherence tomography (OCT)-derived morphologic retinal features have been introduced.^3^^–^^5^

The primary objective of the PINNACLE study is to refine morphologic markers and endpoints further. However, despite the quantitative advantages of morphologic metrics, regulatory agencies have stressed the importance of functional outcome measures, as they confirm relevance to patients.^6^ Best-corrected visual acuity (BCVA) has predominantly been employed as a functional endpoint in clinical trials for macular diseases, but BCVA is not always sensitive to disease progression.^7^ It is primarily a readout of foveal function and unsuitable for evaluating parafoveal or outer macular performance, which is a drawback in assessing extrafoveal lesions.^8^ Also, the heterogeneous photoreceptor composition between the foveal and parafoveal region and their distinct susceptibility to disease^9^ warrants using a more wide-ranging functional outcome measure. Specifically, it has been shown that rod loss, a hallmark of aging and AMD, is most pronounced at 5° eccentricity from the foveal center.^10^^,^^11^

An outcome measure, which not only evaluates foveal but macular visual performance, is fundus-controlled perimetry or microperimetry (MP). Modern MP devices like the MAIA (CentreVue, Padova, Italy) facilitate the spatial coregistration of visual sensitivity test points to a fundus image through eye tracking.^12^^,^^13^ MP has been reported to be an informative metric in AMD^14^^,^^15^ with significant correlations to morphologic metrics.^16^^–^^21^ However, current MP-derived metrics have been shown to have a high test–retest variability^22^^,^^23^ and exhibit low sensitivity toward the onset of advanced AMD.^18^ Therefore, the validation of novel MP-derived metrics is essential. Weleber et al.^24^ and Josan et al.^25^ recently introduced custom software to obtain three-dimensional (3D) hill-of-vision (HOV) surface plots and the total volume (VTOT) beneath the plots from MP raw data. These volumetric visual field interpolation approaches have been described to yield fine-grained retinal sensitivity metrics in the context of inherited retinal degenerations.^26^^–^^28^ To our best knowledge, these new metrics have so far not been tested in the context of iAMD, and there is a lack of comparison between the two available software applications. Further, apart from the MACUSTAR study,^29^ there is a shortage of large-scale multicenter visual field data in iAMD.

Accordingly, we investigated the cross-sectional associations of MP retinal sensitivity and fixation stability metrics with demographic features as well as with BCVA and low-luminance visual acuity (LLVA), utilizing baseline data from the large prospective PINNACLE study cohort of patients with iAMD. Additionally, we compare the published HOV MP metrics of Weleber et al.^24^ and Josan et al.^25^ in the context of iAMD.

## Methods

The PINNACLE study (ClinicalTrials.gov NCT04269304) protocol was approved in the United Kingdom by the East Midlands–Leicester Central Research Ethics Committee (ref. 19/EM/0163) and further by the institutional review boards of all involved institutions. It adheres to the principles of Good Clinical Practice and is in accordance with the Declaration of Helsinki. Informed consent was obtained from the participants after explanation of the nature and possible consequences of the study. PINNACLE is a multicenter, noninterventional prospective predictive modeling study with a retrospective data study running in parallel. Details on design, inclusion and exclusion criteria, organization, data collection, and management are outlined in the PINNACLE trial protocol.^30^ The grading of AMD stages was conducted according to the Beckman classification,^2^ with iAMD defined by large drusen (>125 µm) and/or any definite hyper- or hypopigmentary abnormalities associated with medium or large drusen. Eyes with complete retinal pigment epithelium (RPE) and outer retinal atrophy^31^ were not included in iAMD. BCVA and LLVA were measured with Early Treatment of Diabetic Retinopathy Study (ETDRS) charts.

### Microperimetry

**Figure 1.**
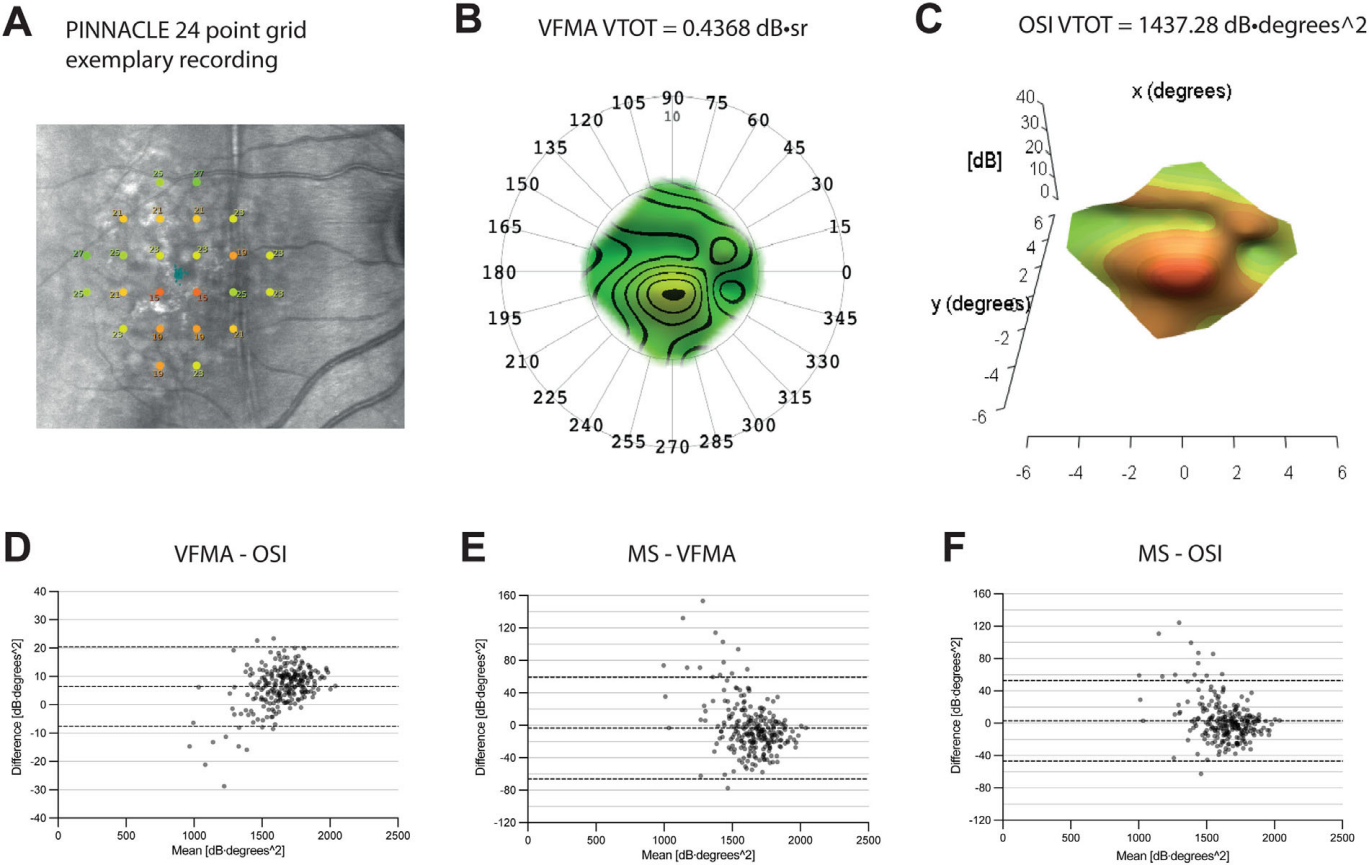
Hill of vision metrics in iAMD. (**A**) PINNACLE 24-point grid exemplary recording. (**B**) Corresponding HOV surface plot derived from the VFMA software. (**C**) Corresponding HOV surface plot derived from the OSI. (**D**) Bland–Altman plot comparing VFMA (converted to dB⋅degrees^2^) and OSI output. (**E**) Bland–Altman plot comparing MS and VFMA output (both converted to dB⋅degrees^2^). (**F**) Bland–Altman plot comparing MS (converted to dB⋅degrees^2^) and OSI output. The bias as the mean of differences (*middle line*) and the 95% limits of agreement (*outer lines*) are shown in all Bland–Altman plots.

All study centers conducted MP measurements using the MAIA device (CentreVue). This machine presents white Goldmann III stimuli (0.43° diameter) and features a maximum luminance of 318.3 cd/m^2^ with a dynamic range of 0 to 36 dB on a background of 1.27 cd/m^2^. In the PINNACLE study, three different MP testing grids are employed as described in the PINNACLE trial protocol.^30^ This study analyzed the 24-point PINNACLE standard grid, run at the baseline visit. The grid is centered in the fovea, and the 24 testing points cover the central 10 degrees in diameter (Fig. 1A). In cartesian coordinates, the test points are located on the −5°, −3°, −1°, 1°, 3°, 5° positions on both the x-axis and the y-axis. A traditional MP-derived metric for retinal sensitivity is mean sensitivity (MS) in dB, which is obtained by averaging all sensitivities of individual test points. Additionally, we applied the software by Weleber et al.^24^ (Visual Field Modeling and Analysis [VFMA]; Scott Gillespie, Applied Brain, Portland, OR, USA; Version 2.0.34 (34), shared under a Collaborative Research Agreement from OHSU, Office of Business Collaboration and Technology Transfer, Portland, OR, USA) and Josan et al.^25^ (open-source wrapper function for thin plate spline interpolation of visual field data in R, open-source implementation [OSI]) to obtain 3D HOV surface plots. The VFMA software uses polar coordinates, whereas the OSI operates with cartesian coordinates. The outcome measure deduced from the surface plot by approximate integration is the total volume underneath the surface (VTOT). The polar unit of the VFMA software is dB⋅sr, as opposed to the cartesian OSI unit of dB⋅degrees^2^. Both units can be converted into each other to facilitate comparisons. Units of VFMA volumes were converted from dB⋅sr to dB⋅degrees^2^ using the conversion 1 sr = (180/π)^2^ ∼ 3282.8 degrees^2^. Units of MS were converted from dB to dB⋅degrees^2^ multiplying by the area of the PINNACLE standard grid (area = 68 degrees^2^). The patients’ fixation stability was reported by the bivariate contour ellipse area encompassing 95% of the fixation measurements (BCEA95).^32^ Further details on MP procedures are outlined in the PINNACLE trial protocol.^30^

### Statistical Analysis

GraphPad Prism (GraphPad Software, San Diego, CA, USA), Microsoft Excel (Microsoft Corporation, Redmond, WA, USA), and the software environment R^33^ with add-on packages lme4, sjPlot, and lmerTest were used for statistical analysis. Data were assessed for Gaussian normal distribution by the D'Agostino and Pearson test. If continuous data were normally distributed, unpaired *t*-test with Welch's correction was used, and in the case of paired data, paired *t*-test was performed. If continuous data were not normally distributed, the Mann–Whitney test was used, and the Wilcoxon matched-pairs signed rank test was conducted in the case of paired data.

Bland–Altman plots were used for methodologic comparison. For univariable association analysis, linear mixed-effects models were used with the respective metric of retinal sensitivity (MS, VFMA VTOT, or OSI VTOT) as the dependent variable. Eyes nested in patients were considered a random-effects term. We examined the following variables as explanatory variables (fixed effects): BCVA, LLVA, BCEA95, lens status, smoking status, and sex.

For variable selection in the multivariable analysis, we performed a backward selection (using *F*-tests of fixed-effect terms) and validated the selection with forward selection (based on the Bayesian information criterion [BIC]). Again, the respective retinal sensitivity metric was considered the dependent variable and eyes nested in patients as the random-effects term.

Hypothesis tests were performed using a 5% (0.05) significance level. For normally distributed data, mean and standard deviation (SD) are presented, and for not normally distributed data, median and interquartile range (IQR) are presented.

## Results

### Cohort Characteristics

This baseline analysis of MP data from the PINNACLE study included 247 study eyes of 189 patients with iAMD. Of the 276 study eyes that underwent the full study protocol, including MP at baseline, 29 eyes had to be excluded from the analysis due to insufficient functional data quality. The mean ± SD age of the participants was 75 ± 7.3 years with 64% females and an almost balanced right-to-left eye ratio (122:125) in a white-dominated study population (white/Asian/black/other, 238/5/2/2). Of the study eyes, 64.8% were phakic and 35.2% pseudophakic, and the smoking status was as follows for the included study eyes: current smoker, 8.1%; ex-smoker (>1 month), 49.8%; and never smoked, 42.1% (Table 1).

The median BCVA and LLVA were 0.02 logarithm of the minimum angle of resolution (logMAR) [−0.06 to 0.1] and 0.32 logMAR [0.22–0.46], and the participants exhibited a median MS of 24.2 dB [22.8–25.8]. The median VTOTs were 0.51 dB⋅sr [0.47–0.54]/1666 dB⋅degrees^2^ [1546–1770] for VFMA and 1656 dB⋅degrees^2^ [1545–1766] as output by the OSI, respectively, and the participants displayed a median fixation stability (log_10_[BCEA 95%]) of −0.81 degrees^2^ [−1.51 to 1.85].

### HOV Metrics in iAMD

In Figure 1, hill-of-vision surface plots created with the VFMA software (Fig. 1B) and the OSI (Fig. 1C) are displayed. In this cross-sectional analysis of PINNACLE baseline data, the output of VFMA software and OSI exhibited a significant difference (*P* < 0.0001, Wilcoxon matched-pairs signed rank test). As can be inferred from the medians (^1^Table 2) and the respective Bland–Altman plot (Fig. 1D), the VFMA software overall yielded higher values for retinal sensitivity than the OSI (bias [95% limits of agreement]: VFMA–OSI: 6.4 dB⋅degrees^2^ [−7.6 to 20.5]). In the evaluation of eyes with lower mean volumes, there was a trend toward lower values as output by the VFMA software.

**Table 1.** Demographic Data of the Study Population

Parameter	Value
No. of patients	189
No. of study eyes	247
Mean (SD) age, y	75 (7.3)
Sex, *n* (%)	
Female	158 (64.0)
Male	89 (36.0)
Eye, *n* (%)	
Right	122 (49.4)
Left	125 (50.6)
Race, *n* (%)	
White	238 (96.4)
Asian	5 (2.0)
Black	2 (0.8)
Other	2 (0.8)
Lens status, *n* (%)	
Phakic	160 (64.8)
Pseudophakic	87 (35.2)
Smoking, *n* (%)	
Current smoker	20 (8.1)
Ex-smoker	123 (49.8)
Never smoked	104 (42.1)

**Table 2.** Reference Data for Multiple Retinal Sensitivity and Fixation Stability Metrics

Parameter	Median [IQR]
BCVA (logMAR)	0.02 [−0.06 to 0.1]
LLVA (logMAR)	0.32 [0.22–0.46]
MS (dB)/(dB⋅degrees^2^)	24.2 [22.8–25.8]/1646 [1550–1754]
VFMA VTOT (dB⋅sr)/(dB⋅degrees^2^)	0.51 [0.47–0.54]/1666 [1546–1770]
OSI VTOT (dB⋅degrees^2^)	1656 [1541–1761]
Log_10_[BCEA95] (degrees^2^)	−0.81 [−1.51 to 1.85]

Review of the respective Bland–Altman plots (Fig. 1E) revealed that VFMA yielded slightly higher values, and OSI (Fig. 1F) produced slightly lower values compared to the output of MS: bias [95% limits of agreement]: MS–VFMA: −3.5 dB⋅degrees^2^ [−66.1 to 59]; MS–OSI: 2.9 dB⋅degrees^2^ [−46.7 to 52.5]. Both volumetric metrics showed outliers with higher or lower retinal sensitivities compared to MS. Detailed evaluation of the respective exams (Supplementary Fig. S1) showed that both volumetric metrics delivered lower total retinal sensitivities compared to MS, if there was a concentration of lower retinal sensitivity test points in the center of the PINNACLE standard grid. And vice versa, both volumetric metrics delivered higher total retinal sensitivities compared to MS, if there was a concentration of lower retinal sensitivity test points in the periphery of the PINNACLE standard grid. This tendency in the relationship to MS was more pronounced in the VFMA output.

### Relationship of Retinal Sensitivity Metrics to BCVA, LLVA, and BCEA95

**Figure 2.**
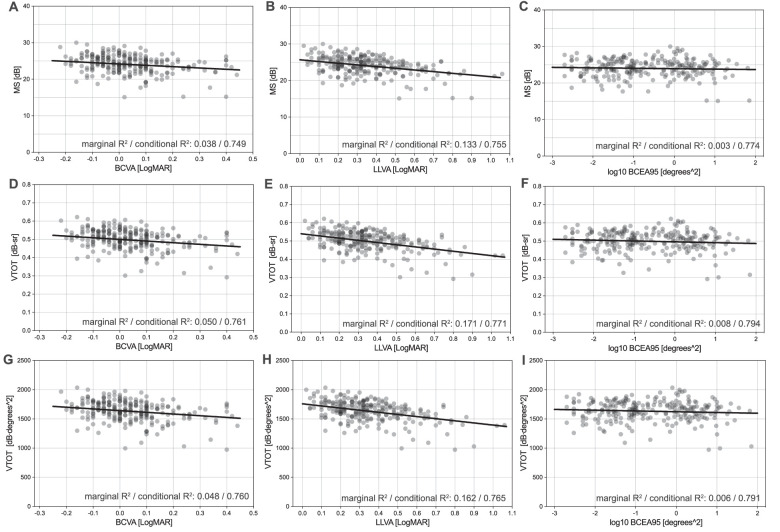
Relationship of retinal sensitivity metrics to BCVA, LLVA, and BCEA95. (**A**–**C**) MS in dB. (**D**–**F**) VFMA VTOT in dB-Sr. (**G**–**I**) OSI VTOT in dB⋅degrees^2^. Univariate linear mixed-model analysis reveals linear relationships for all plotted metrics. Regression lines are plotted. Overall, the models including VFMA VTOT as a fixed effect exhibit the highest values for coefficients of determination: marginal *R*^2^ (variance explained only by fixed effects) and conditional *R*^2^ (variance explained by both fixed and random effects).

Univariate linear regression analysis revealed linear relationships of all tested metrics of retinal sensitivity to BCVA, LLVA, and BCEA95 (Fig. 2). MS exhibited the following associations (slope [95% confidence interval]) to BCVA (−3.63 dB/logMAR [−5.6 to −1.67]), to LLVA (−4.61 dB/logMAR [−6.0 to −3.22]), and to BCEA95 (−0.12 dB/log_10_(degrees^2^) [−0.44 to 0.19]). VFMA VTOT showed the following associations to BCVA (−0.09 dB⋅sr/logMAR [−0.14 to −0.05]), to LLVA (−0.12 dB⋅sr/logMAR [−0.15 to −0.09]), and to BCEA95 (−0.005 dB⋅sr/log_10_(degrees^2^) [−0.01 to −0.001]). And finally, OSI VTOT displayed the following associations to BCVA (−290.84 dB⋅degrees^2^/logMAR [−429.83 to −151.86]), to LLVA (−363.64 dB⋅degrees^2^/logMAR [−461.19 to −266.09]), and to BCEA95 (−13.14 dB⋅degrees^2^/log_10_(degrees^2^) [−35.71 to 9.42]). See Supplementary Table S1 for a full list of mixed-model intercept, slope, marginal *R*^2^, and conditional *R*^2^. LLVA exhibited the strongest associations, and the fixation stability metric BCEA95 showed the weakest associations with retinal sensitivity metrics. Further, the models with VFMA VTOT as the dependent variable consistently exhibited slightly higher coefficients of determination (both marginal *R*^2^ and conditional *R*^2^) compared to the models with MS and OSI VTOT as fixed effects (Fig. 2, Supplementary Table S1).

### Relationship of Retinal Sensitivity Metrics to Lens Status, Smoking History, and Sex

**Figure 3.**
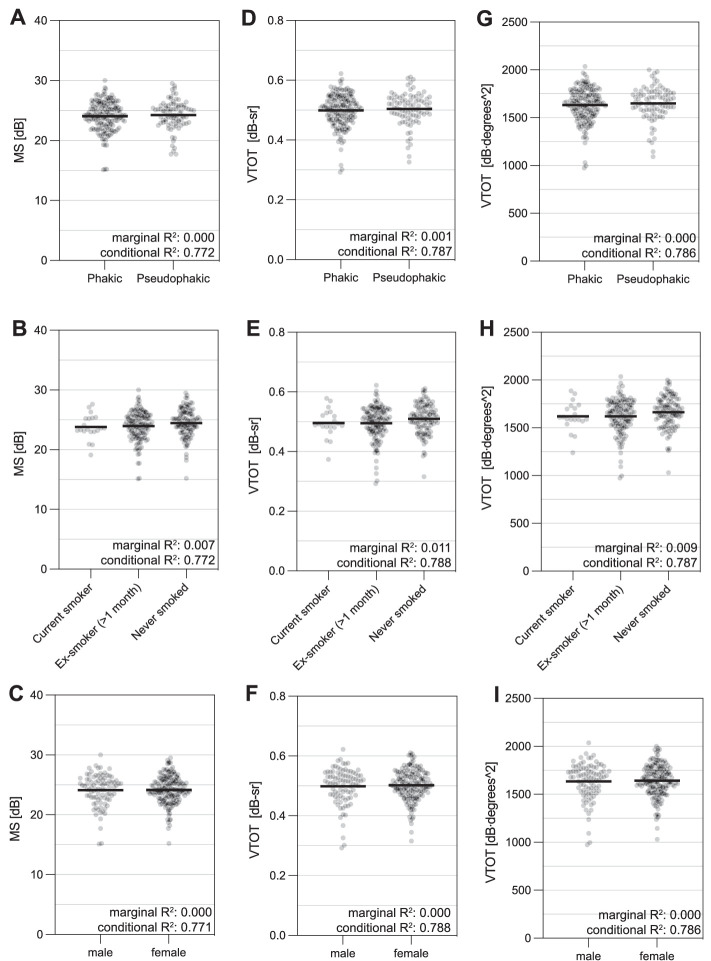
Relationship of retinal sensitivity metrics to lens status, smoking history, and sex. (**A**–**C**) MS in dB. (**D**–**F**) VFMA VTOT in dB-Sr. (**G**–**I**) OSI VTOT in dB⋅degrees^2^. In univariate linear mixed-model analysis, models including VFMA VTOT as a fixed effect yield the highest coefficients of determination. None of the demographic variables shows a significant association with the retinal sensitivity metrics, even though there was a trend for VFMA VTOT/never smoked (*P* = 0.3). The mean is plotted (*black bar*).

Univariate linear regression analysis did not show significant associations between retinal sensitivity metrics and lens status, smoking, and sex (Fig. 3). As with the visual acuity and fixation variables described above, the models including VFMA VTOT consistently exhibited slightly higher coefficients of determination (both marginal *R*^2^ and conditional *R*^2^) compared to the models with MS and OSI VTOT as fixed effects. See Supplementary Table S2 for a full list of mixed model estimate, marginal *R*^2^, and conditional *R*^2^.

### Multivariable Analysis of Associations to Retinal Sensitivity

**Figure 4.**
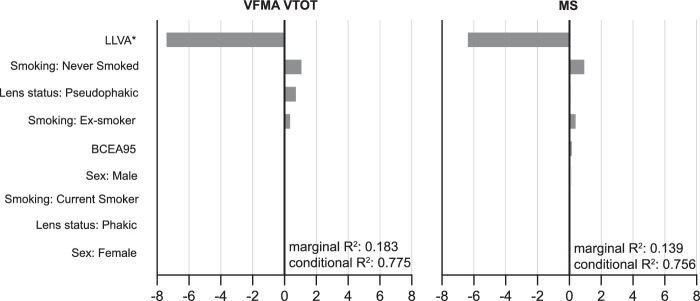
Variable importance for VFMA VTOT and MS. Multivariable linear mixed-effect model with all candidate variables included. The variable importance is shown as a *t*-statistic. *Please note that BCVA and LLVA showed a strong correlation, resulting in model overfitting when including both variables. LLVA was the only demonstrable predictor of retinal sensitivity and hence was selected over BCVA to be plotted as visual acuity in this figure.

In the multivariable analysis (Fig. 4), LLVA showed the strongest association with and was the only demonstrable predictor of VFMA VTOT (*t*-value, *P*-value: −7.5, <0.001) and MS (−6.5, <0.001). The multivariable model with VFMA VTOT (marginal *R*^2^/conditional *R*^2^: 0.183/0.775) as the dependent variable displayed slightly higher coefficients of determination than the model with MS (0.139/0.756).

## Discussion

This cross-sectional analysis of PINNACLE baseline data explored different, including novel, MP-derived retinal sensitivity and fixation stability metrics and their associations with visual acuity measures and demographic features in iAMD.

This study cohort's median MS [IQR] was 24.2 [22.8–25.8] dB. There are several smaller published studies measuring baseline MS in iAMD with the MAIA device: Roh et al.^21^ (Age-Related Eye Disease Study (AREDS) 2001 classification, *n* = 71, mean age = 69.7 years, mean ± SD MS: 26.0 ± 4.3 dB), Vujosevic et al.^14^ (AREDS 2001 classification, *n* = 12, mean age = 72 years, mean ± SD MS: 26.2 ± 2.3 dB), Wu et al.^34^ (Beckmann classification, *n* = 41 [stable/progressed], mean age = 68.8 years, mean ± standard error MS: 26.6 ± 0.17 dB). All of these studies report higher values for MS, which could be linked to the lower mean age of these study cohorts compared to the PINNACLE study (75 years), as retinal sensitivity has been described to be significantly negatively associated with age in normal aging^35^^,^^36^ and also specifically in AMD,^21^ which also holds true for the PINNACLE data. The use of the AREDS 2001^37^ classification in Roh et al.^21^ and Vujosevic et al.^14^ for iAMD as opposed to the use of the Beckmann^2^ classification in the PINNACLE study could also have contributed to the difference. With regard to visual acuity (PINNACLE median [IQR]: BCVA 0.02 [−0.06-0.1], LLVA 0.32 [0.22–0.46]), the respective studies report parametric statistics (mean [SD]), which for Vujosevic et al.^14^ (BCVA 0.093 ± 0.14) and Roh et al.^21^ (BCVA 0.09 ± 0.12) convey worse and for Wu et al.^34^ (BCVA ∼−0.01, LLVA ∼0.3) comparable performance to the PINNACLE study cohort.

In contrast to visual acuity, MP facilitates the evaluation of visual performance not only in the fovea but throughout the macula. Thereby, it tests visual performance in regions, which cannot be tested by reading letter charts but also meaningfully contribute to activities of daily living.^38^^,^^39^ Evaluating longitudinal MP changes in MS has been reported to be more valid than follow-ups of individual test points.^22^ At the same time, MS and mean deviation (MD) are based on averaging the recorded sensitivity values.^40^ Thus, these metrics are applicable for rectilinear grids with equally spaced stimuli and should only be used with caution interpreting grids with radial patterns and unequal spacing.^24^ Contrarily, with the 3D HOV surface plots and the derived metrics (e.g., VTOT), the spatial circumstances of retinal sensitivity are taken into account, and therefore even comparison among grids with different sampling patterns is feasible. In this analysis of baseline PINNACLE MP data, we found that in exams with a concentration of lower retinal sensitivity in the center of the PINNACLE standard grid, both volumetric metrics delivered lower total retinal sensitivities compared to MS. Also contrarily, higher total retinal sensitivities were delivered in exams with a concentration of lower retinal sensitivity in the periphery (Supplementary Fig. S1). These findings suggest that in volume calculations, both volumetric methods convey a higher weight to the central test points as opposed to the peripheral test points of the evaluated MP grid. Yet, compared to MS, VTOT derived from both tested custom software applications yielded slightly higher coefficients of determination in association with commonly used variables as determined with linear mixed-model analysis (Figs. 2^3^–4). As indicated above, volumetric measures have been described to be particularly advantageous over MS and MD comparing grids with radial patterns or unequal spacing. However, the PINNACLE standard grid is a rectilinear grid with evenly spaced stimuli, which favors the MS representation. Therefore, it makes sense that in the analysis at hand, volumetric measures do not yield markedly different evaluations of retinal sensitivity than MS. Moreover, macular retinal sensitivities in an iAMD population are not as heterogeneous as in advanced retinal disease. Nonetheless, in the trajectory toward advanced AMD, more heterogeneity can be expected, and in such conditions, volumetric measures are reported to be more precise than MS.^25^ It is therefore sensible to characterize retinal sensitivity with volumetric measures in iAMD to consistently describe the functional trajectory toward advanced AMD. Finally, it has to be mentioned that more elaborate HOV-based metrics are currently evolving, which could, together with VTOT, be potential endpoints for therapeutic trials.

Comparing the performance of VFMA and OSI software applications in the context of iAMD, we found a significant but small difference between the output of the two applications. Further, the Bland–Altman plot analysis of VFMA and OSI output showed that VFMA generally yielded higher values than OSI. Finally, our data suggest that in the volume calculation, VFMA compared to OSI conveys a higher weight to the central test points as opposed to the peripheral test points of the evaluated MP grid. Josan et al.^25^ also compared their OSI output and the VFMA output on nine MAIA recordings of eyes with different macular diseases and of one healthy control. Based on the calculation of the intraclass correlation coefficient from this sample, the authors conclude that there is an excellent agreement between the two metrics. Nevertheless, on account of our data, the output of VFMA and OSI is not exactly interchangeable.

Interestingly, VFMA VTOT as the dependent variable yielded slightly higher coefficients of determination in association with other variables as determined with linear mixed-model analysis than with OSI VTOT (Figs. 2^3^–4). Longitudinal analysis will be necessary to assess whether one interpolation approach outperforms the other regarding the ability-to-detect change.

It has been previously reported that cataract can significantly impact MP results.^41^ Our data provide no association between retinal sensitivity and lens status in the PINNACLE study cohort. This is likely attributable to the study inclusion criteria,^30^ limiting the range of lenticular opacification. Moreover, our data do not demonstrate significant associations between smoking status and retinal sensitivity. Yet, there is a trend toward higher retinal sensitivities in the eyes of participants who have never smoked, compared to current or ex-smokers (Fig. 3). Smoking is a known risk factor for the onset and progression of AMD.^42^^–^^44^

In univariable analysis, our data provide significant associations of both BCVA and LLVA to the tested metrics of retinal sensitivity. At the same time, the univariable models, including LLVA, consistently offered the highest coefficients of determination signifying the best model fitting. In multivariable analysis, the strong correlation of BCVA and LLVA led to overfitting when including both variables in the model. Backward variable selection and subsequent validation by forward selection based on the BIC eliminated BCVA from the model, leaving LLVA as the only demonstrable predictor of retinal sensitivity. However, due to their strong correlation, LLVA can be considered to illustrate BCVA in this multivariable model indirectly. These findings are in accordance with Wu et al.,^45^ who also found retinal sensitivity to have a stronger association with LLVA than with BCVA in a cross-sectional study of 179 participants of different AMD stages. Wu et al.^45^ suggest that LLVA may capture a greater extent of a foveal functional deficit than BCVA, while pointing out that in their study, which contained a group of healthy controls, MP-derived MS was more sensitive to foveal functional deficiency than both measures of visual acuity.

The following limitations to this analysis of iAMD MP baseline data of the PINNACLE study need to be considered. First, the study population is almost exclusively composed of white Europeans. Therefore, findings from this analysis might not be generalizable to other ethnicities. Second, this study does not include age-matched healthy controls; therefore, the sensitivity of different metrics toward macular functional deficits compared to control cannot be evaluated. Third, this is a cross-sectional analysis; hence, from this analysis, no inference on the capacities of MP metrics to describe or predict disease progression can be deduced.

In summary, we report reference data for multiple retinal sensitivity and fixation stability metrics in a large cohort of patients with iAMD. Further, for the first time, we utilize the HOV-derived metric of VTOT to evaluate retinal sensitivity in iAMD and demonstrate its favorable characteristics compared to MS. In our data set, VFMA and OSI, the two available HOV software applications, produced similar but statistically significantly different outputs for VTOT. Accordingly, the methods are not exactly interchangeable. VFMA-based VTOT showed a slightly stronger association with other visual function tests and patient characteristics. In general, we attribute great potential to HOV-derived metrics as endpoints for therapeutic trials and encourage the further improvement of OSIs, which make these more accessible.

## Supplementary Material

Supplement 1

Supplement 2
