# Supplementary material for: Comparison of Novel Volumetric Microperimetry Metrics in Intermediate Age-Related Macular Degeneration: PINNACLE Study Report 3
Source: Transl Vis Sci Technol. 2023 Aug 25;12(8):21. doi: 10.1167/tvst.12.8.21 (PMC10461689; doi:10.1167/tvst.12.8.21)
Supplement: Supplement 1 [file tvst-12-8-21_s001.pdf]

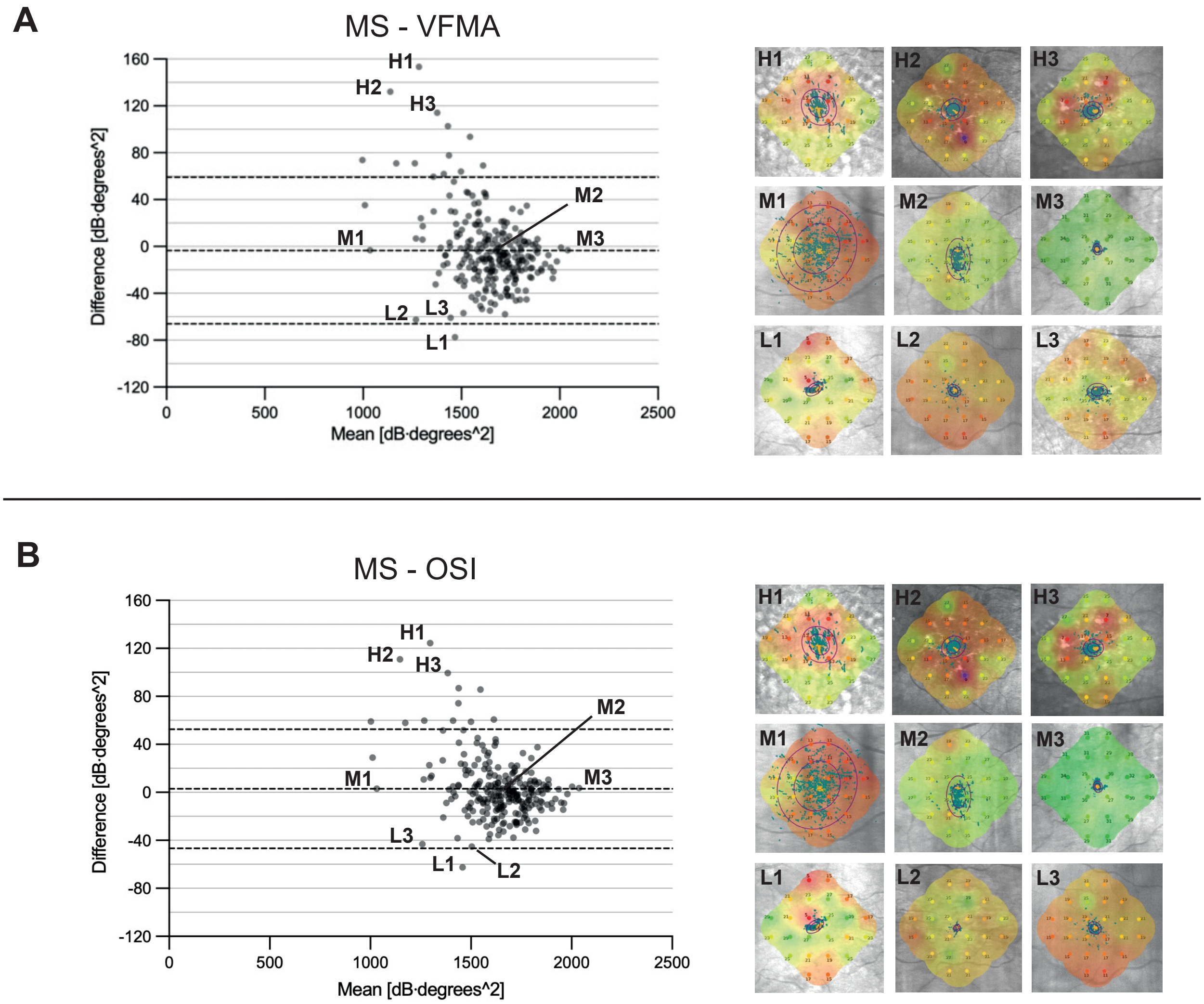

Figure S1. Hill-of-vision metrics in iAMD

A) Bland-Altman comparison of MS and VFMA output. Exams for positive (H), negative (L) and small (M) differences in retinal sensitivities are highlighted within the Bland-Altman plot and the respective microperimetry pointwise retinal sensitivities blended on SLO macular images are displayed.

B) Bland-Altman comparison of MS and OSI output. Exams for positive (H), negative (L) and small (M) differences in retinal sensitivities are highlighted within the Bland-Altman plot and the respective microperimetry pointwise retinal sensitivities blended on SLO macular images are displayed.
