# Supplementary material for: Comparison of Novel Volumetric Microperimetry Metrics in Intermediate Age-Related Macular Degeneration: PINNACLE Study Report 3
Source: Transl Vis Sci Technol. 2023 Aug 25;12(8):21. doi: 10.1167/tvst.12.8.21 (PMC10461689; doi:10.1167/tvst.12.8.21)
Supplement: Supplement 2 [file tvst-12-8-21_s002.pdf]

Table S1. Relationship of retinal sensitivity metrics to BCVA, LLVA and BCEA95

mixed model intercept [95% CI]	slope [95% CI]	marginal R^2	conditional R^2
**Association of MS with BCVA**			
24.19 dB [23.85 to 24.52]	-3.63 dB/logMAR [-5.6 to -1.67]	0.038	0.749
**Association of MS with LLVA**			
25.67 dB [25.09 to 26.24]	-4.61 dB/logMAR [-6.0 to -3.22]	0.133	0.755
**Association of MS with BCEA95**			
23.98 dB [23.58 to 24.38]	-0.12 dB/log10(degrees^2) [-0.44 to 0.19]	0.003	0.774
**Association of VFMA VTOT with BCVA**			
0.50 dB·sr [0.49 to 0.51]	-0.09 dB·sr/logMAR [-0.14 to -0.05]	0.050	0.761
**Association of VFMA VTOT with LLVA**			
0.54 dB·sr [0.53 to 0.55]	-0.12 dB·sr/logMAR [-0.15 to -0.09]	0.171	0.771
**Association of VFMA VTOT with BCEA95**			
0.50 dB·sr [0.49 to 0.51]	-0.005 dB·sr/log10(degrees^2) [-0.01 to -0.001]	0.008	0.794
**Association of OSI VTOT with BCVA**			
1642.68 dB·degrees^2 [1618.53 to 1666.83]	-290.84 dB·degrees^2/logMAR [-429.83 to -151.86]	0.048	0.760
**Association of OSI VTOT with LLVA**			
1759.51 dB·degrees^2 [1719.13 to 1799.9]	-363.64 dB·degrees^2/logMAR [-461.19 to -266.09]	0.162	0.765
**Association of OSI VTOT with BCEA95**			
1624.44 dB·degrees^2 [1595.76 to 1653.13]	-13.14 dB·degrees^2/log10(degrees^2) [-35.71 to 9.42]	0.006	0.791

Table S2. Relationship of retinal sensitivity metrics to lens status, smoking history and sex

Variable	Mixed model estimate [95% CI]	marginal R^2	conditional R^2
**Association of MS with lens status**		0.000	0.772
**Phakic**	24.08 dB [23.66 to 24.51]		
**Pseudophakic**	24.02 dB [22.93 to 25.11]		
**Association of MS with smoking**		0.007	0.772
**Current Smoker**	23.61 dB [22.36 to 24.87]		
**Ex-Smoker**	23.96 dB [21.36 to 26.56]		
**Never smoked**	24.26 dB [21.65 to 26.89]		
**Association of MS with sex**		0.000	0.771
**female**	24.05 dB [23.62 to 24.49]		
**male**	24.07 dB·sr [22.92 to 25.22]		
**Association of VFMA VTOT with lens status**		0.001	0.787
**Phakic**	0.50 dB·sr [0.49 to 0.51]		
**Pseudophakic**	0.50 dB·sr [0.48 to 0.53]		
**Association of VFMA VTOT with smoking**		0.011	0.788
**Current Smoker**	0.49 dB·sr [0.46 to 0.52]		
**Ex-Smoker**	0.5 dB·sr [0.44 to 0.56]		
**Never smoked**	0.51 dB·sr [0.45 to 0.57]		
**Association of VFMA VTOT with sex**		0.000	0.788
**female**	0.5 dB·sr [0.49 to 0.51]		
**male**	0.5 dB·sr [0.47 to 0.52]		

Variable	Mixed model estimate [95% CI]	marginal R^2	conditional R^2
**Association of OSI VTOT with lens status**		0.000	0.786
**Phakic**	1633.66 dB·degrees^2 [1603.26 to 1664.05]		
**Pseudophakic**	1630.97 dB·degrees^2 [1552.77 to 1709.16]		
**Association of OSI VTOT with smoking**		0.009	0.787
**Current Smoker**	1600.87 dB·degrees^2 [1510.32 to 1691.42]		
**Ex-Smoker**	1621.78 dB·degrees^2 [1434.14 to 1809.42]		
**Never smoked**	1651.69 dB·degrees^2 [1462.72 to 1840.66]		
**Association of OSI VTOT with sex**		0.000	0.786
**female**	1634.45 dB·degrees^2 [1603.02 to 1665.87]		
**male**	1629.65 dB·degrees^2 [1546.39 to 1712.89]		
